# Development, Physicochemical, and Sensory Evaluation of Naturally Formulated Dehydrated Fruits Rolls Enriched with Sea Buckthorn and Grape Freeze-Dried Pomace Powders

**DOI:** 10.3390/foods15173059

**Published:** 2026-08-28

**Authors:** Luminița Grosu, Oana-Irina Patriciu, Irina-Claudia Alexa, Adriana-Luminița Fînaru

**Affiliations:** Department of Chemical and Food Engineering, Faculty of Engineering, “Vasile Alecsandri” University of Bacău, 157, Calea Mărăşeşti, 600115 Bacău, Romania; lumig@ub.ro (L.G.); oana.patriciu@ub.ro (O.-I.P.); adrianaf@ub.ro (A.-L.F.)

**Keywords:** pomace, fruit processing, dehydrated fruit rolls, bioactive compounds, sensory study, global satisfaction, food sustainability

## Abstract

Pomaces, by-products from beverage industries, which are extremely rich in various bioactive compounds such as dietary fiber, polyphenolic compounds, carotenoids, etc., represent valuable sources of natural antioxidants. Due to their nutritional properties, innovative food products could be developed by incorporating pomace powders as potentially functional ingredients. In the present work, new dehydrated fruit-based products, known as fruit rolls or leathers, were developed from apples and bananas (2/1, *w*/*w*), with honey (2%), lemon juice (2%), and freeze-dried grape or sea buckthorn pomace powders (3%), without artificial sweeteners, preservatives, or hydrocolloids. Several physicochemical (moisture content, water activity, pH, CIE L*a*b*) and chemical (total phenolic and carotenoid contents) parameters were investigated both for pomace powders and developed fruit rolls. Moreover, the prepared fruit rolls were evaluated in terms of sensorial characteristics using a seven-point hedonic scale. The samples obtained by incorporation of freeze-dried pomace powders exhibited increased values of the total phenolic content (86.54 and 95.77 mg GAE/g d.m.) compared to the control sample (59.62 mg GAE/g d.m.). Sensory evaluation with twenty untrained panelists indicated good acceptability, with mean scores above 6.00. The addition of freeze-dried pomace powders improved the visual attributes, the appearance and color of the corresponding fruit rolls receiving slightly higher scores compared to the control. The newly developed fruit rolls with freeze-dried grape or sea buckthorn pomace powders could represent a tasty, interesting alternative for sweet snacks.

## 1. Introduction

Rapid growth in the world’s population and changes in lifestyle have led to a significant increase in food waste from various industrial, agricultural, and domestic sources [1,2].

Agri-food by-products and waste contain high amounts of organic matter, which, if not managed correctly, can pose a serious threat to both the environment and human health; thus, the changeover from simple disposal to strategic food waste management is essential. Education about the economic and environmental impact of food waste or by-products plays an important role in the involvement and efforts of the young generation to reduce food waste [2,3].

Various types of food waste or by-products, such as waste from fruits, vegetables, grains, and other food production and processing products, contain important bioactive compounds, such as polyphenols, fibers, proteins, lipids, vitamins, organic acids, and minerals, some of which are found in large quantities. These bioactive compounds offer the potential to convert by-products and waste of agro-food origin into valuable products, in areas such as nutritional foods, biofertilizers, bioenergy, biosurfactants, etc. [2,3,4,5].

Moreover, the consumer has increased interest in diet and health, demanding suppliers consider the reuse of agro-food by-products. Thus, the application of green recovery technologies (e.g., ultrasound, microwave, and enzyme-assisted extraction) eliminates the harmful effects comparing to conventional technologies (e.g., maceration or Soxhlet extraction using toxic organic solvents), and waste can be recycled into the food chain as functional additives for different products and applications, guaranteeing the sustainability and reducing the food by-products. Over the last decade, significant research attention in food fields has focused on addressing the current market demands of consumers by creating new, sustainable, alternative, healthier foods. For this reason, the application of a circular economy in food systems represents a key strategy for the future [1,2].

Interest in investigations concerning the reuse of by-products and waste of agro-food origin is increasing, with the aim of finding innovative solutions to value them in different areas [5,6]. The alcoholic and non-alcoholic beverages industry represents one of the industries from which a large amount of by-products results. Pomace is a solid residue consisting of a mixture of skins, seeds, and any other solid remaining after pressing from juice or wine manufacturing. It is well known that due to their important bioactive compound content (polyphenols, organic acids, minerals, etc.), the pomaces possess remarkable antioxidant and antibacterial properties [7,8,9,10].

According to the International Organization of Vine and Wine, Romania is an important wine producer, ranking 13th globally in 2025 [11]. In this context, the concern for reducing waste from the wine industry represents a significant component of environmental policy at the national level, namely finding new solutions for the recovery of pomace, which is extremely rich in dietary fiber, lipids, and polyphenolic compounds [12,13,14,15,16,17]. *Fetească neagră* (Black Maiden) is a dark-skinned grape variety of Romanian grape cultivated mainly in several regions, including Moldova. In 2020, the most consumed wine variety in Romania was the one obtained from this variety of grapes [18]. Grape pomace from Romanian red variety of *Vitis vinifera* L. var. *Fetească neagră* is reported as an important source of natural antioxidants, because of its high concentration of different phenolic compounds that possess antioxidant and cardioprotective effects [19].

On the other hand, the sea buckthorn (*Hippophae rhamnoides* L.) is a very well-known berry for multiple health benefits due to its bioactive compounds, consisting of carotenoids and polyphenols with important antioxidant characteristics, highly appreciated by Romanian consumers. In Romania, the sea buckthorn grows wild in the sub-Carpathian area of Moldova and Muntenia, from the Siret basin to Olt River. In recent years, due to consumer interest in the products obtained from these fruits, more and more plantations have been developing. Sea buckthorn fruits are less consumed as such due to their astringent taste, but they can be found in various products: juice, jellies, liqueurs, sweets, vitamin C tablets, oil, food pigment, jam, cookies, etc. Large amounts of sea buckthorn pomace are produced during juice extraction. Due to its variety of biologically active compounds, this pomace can be further processed and used [8,20,21,22,23]. To reduce waste, in recent years, grape pomace and sea buckthorn pomaces are generally used as animal feed, for the extraction of biologically active compounds, or the development of functional foods and dietary supplements [24,25,26,27,28].

Fruit rolls, also known, as fruit leathers are dehydrated fruit-based products, often eaten as a snack or dessert, which can represent an interesting alternative to replace conventional candies or dried fruits [29,30,31,32]. Considered a healthy food, fruit rolls are very well known in the USA, being one of the most popular dried food snacks, while in Europe they are not so popular. Most fruit leathers are obtained by mixing fruit puree and other ingredients or additives like sugar, pectin, citric acid, and glucose syrup and then dehydrating it under specific conditions [33,34,35,36,37]. Fruit rolls usually have intermediate water activity, low moisture content, and low pH, resulting in high microbial stability during storage.

Another important aspect that should be mentioned is that for the preparation of fruits rolls, fruits with external defects may be used, contributing once again to avoiding food waste. It is well-known that, during the long marketing chain of fruits, physical damage of fruits can appear. The external aspect of fruits plays a central role in the definition of quality standards. Some defects may affect the consumers’ quality perceptions, making fruits not suitable for sale in supermarkets [37,38]. Dehydration of fruits into leather allows them to reduce these losses and extend their shelf life [37,39].

Several studies refer to formulations of various fruit leathers by incorporating plant-based ingredients (concentrates or powder) rich in bioactive compounds such as polyphenols with the aim of increasing their value and obtaining a more attractive color as well [40,41,42,43]. While previous investigations have explored the addition of various pomace powders especially in bakery [24,25,26,27,28], dairy [44] products, and confectionary [45,46], studies focusing on their incorporation into fruit leathers remain limited, as most of them rely on the use of pectin or sugar to achieve the desired texture and sweetness [47,48,49,50,51]. At the same time, consumer demand for preservative-free and clearly labeled functional foods remains a priority for the food industry, and fruit leathers could be a suitable matrix in this regard [39,52,53].

Although various fruit combinations have been explored [52,54,55,56,57,58], the specific apple–banana association is rarely studied [39].

To address these research gaps and support a circular bioeconomy, this study builds upon our ongoing research in this field [59,60] by aiming to develop innovative, sensory-acceptable dehydrated fruit rolls enriched with freeze-dried grape or sea buckthorn pomace powders. To the best of our knowledge, the integration of grape or sea buckthorn pomaces within a combined apple–banana base matrix is particularly sparse. Furthermore, unlike conventional formulations that frequently rely on structural additives or synthetic stabilizers, the present study proposes a completely clean-label formulation utilizing solely honey and lemon juice to optimize the physicochemical and sensory attributes (enhance the flavor and maintain the attractive color of the final product).

This study was designed as a foundational step in a multi-stage product development and valorization project. Our primary objective at this initial phase was to establish a sensorial appealing and feasible baseline recipe that successfully integrates fruit processing side-streams (grape and sea buckthorn pomace) with a good acceptance of panelists since the perception is critical in by-product valorization, regardless of nutritional merit. Alongside the sensory profiling (appearance, color, taste-aroma, sweetness, and texture), and several parameters (such as moisture, pH, water activity, titratable acidity, CIE L*a*b* values), some important bioactive compounds, specifically total polyphenols content (TPC) and carotenoids were determined to verify the retention and enhancement of components derived from the pomace additives.

While these results establish a solid foundation for by-product valorization, product’s antioxidant activity profiling, the active ingredients quantification or microbiological screening represent necessary next steps that will be addressed in future research. Thus, this study provides a framework for further investigations into the storage stability and nutritional quality necessary to determine the commercial feasibility of the developed valorized fruit rolls.

## 2. Materials and Methods

### 2.1. Materials

For the formulation of fruit rolls, apples, bananas (classic yellow Cavendish variety), acacia honey, and lemon (Verna variety) were purchased from a local market in Bacău city (Romania) and stored at 4 °C until the preparation of samples.

Starting from the idea of using local products, apples from the *Idared* variety (a sour apple, widely cultivated in Romania, e.g., Sârca—Iași County), which have a lower-than-average sugar content, were chosen in this study [61].

The grape pomace from Romanian red variety of *Vitis vinifera* (L.) var. *Fetească neagră* was provided by a wine producer (Moșia Panciu, Vrancea County, Romania) and frozen (−18 °C) until further use.

The sea buckthorn pomace (*Hippophae rhamnoides* L.) from an organic orchard was provided by a local producer of juice and oil sea buckthorn (Elexius, Bacău County, Romania) and stored in freezer (−18 °C) until the fruit rolls preparation.

### 2.2. Obtention of Freeze-Dried Pomace Powders

In order to preserve the color and the bioactive compounds content of the grape and sea buckthorn pomaces, a freeze-drying process was applied. The frozen pomaces removed from the freezer were first subjected to a pre-freezing process using Biobase laboratory freeze dryer (model BK-FD12P, Biometch Co., Ltd., Jinan, Shandong, China) for 4 h at −40 °C. The lyophilization was performed in the same apparatus for 24 h (−80 °C, maximum vacuum < 5 Pa).

The following stages involved the grinding of the lyophilized pomace in a household electric grinder (Heinner, model HCG-150SS, 150W, Heinner, Bucharest, Romania) until a homogeneous fine powder was obtained, followed by its sieving, using a digital electromagnetic sieve shaker (Filtra Vibración, model IRIS FTS-0200, Barcelona, Spain).

Only the fractions not exceeding 0.500 mm were accepted for further incorporation in the fruit puree. The freeze-dried powders were preserved at room temperature in hermetically sealed bags, in the absence of light and humidity. The abbreviation used further in the manuscript is FD-GPP for the freeze-dried grape pomace powder and FD-SBPP for the freeze-dried sea buckthorn pomace powder.

### 2.3. Preparation of Fruits Rolls Enriched with Freeze-Dried Pomace Powders

The first step in the preparation process was washing the apples, bananas, and lemons to avoid the risk of any kind of contamination. In order to obtain a finished product with uniform properties, both within the same batch and from one batch to another, it was necessary to remove the skin and seeds of the apples. Most formulations in published research have used fruit peeling [41,47]. The decision to peel the fruits was not a waste-generating step, but rather part of a multi-stage circular economy approach. Firstly, the raw materials used in this study were surplus apples presenting aesthetic defects, skin blemishes, or physical bruising. These fruits are typically rejected by the fresh market and destined for disposal; thus, utilizing them directly addresses food loss prevention. Secondly, the removed peels were not discarded as waste. Instead, they were collected and dried with the intention of repurposing as a raw material for developing organic biofertilizers. Therefore, instead of breaking the circular economy concept, this process showcases a cascading valorization strategy: the flesh is upcycled into dehydrated fruit rolls, while the by-product (the peel) is diverted from landfills to agricultural soil amendment. Valorization of different by-products into biofertilizers follows our department’s ongoing framework for food industry waste reduction, as recently demonstrated in our study [62]. At the same time, the skins can complicate the homogenization process of the fruit puree and affect the fine characteristics of the fruit rolls, while the seeds run the risk of releasing unpleasant flavoring substances. The bananas were also peeled. The additional step consists of cutting the fruits into small pieces to facilitate the subsequent mixing and homogenizing steps.

The fresh lemon juice was obtained just before the process of mixing all the ingredients using a household electric citrus-juicer (Black&Decker Model CJ700N-QS, Towson, MD, USA). Lemon juice, together with honey, also facilitates the process of mixing the raw materials by providing additional moisture, thus reducing the need to add water.

In the first stage, in order to find the optimal formulation, several trials were carried out (Table S1 from Supplementary Materials). Initial tests were conducted by varying the proportions of ingredients followed by an informal sensory evaluation focusing on attributes such as taste, texture, color, etc. The final formulation was selected because it represented the optimal balance of sweet–sour flavor, attractive color, and good taste. Thus, the proportions for all ingredients were established: the ratio of peeled apples: bananas was set at 2/1 (*w*/*w*). Honey (2%, *w*/*w*), lemon juice (2%, *w*/*w*), and freeze-dried pomace powder (3%, *w*/*w*) were added to the resulting puree mass. Batches of 1000 g of apple and banana puree were prepared: a control sample (without any pomace powder) and two with the incorporation of sea buckthorn and grape pomace powder, respectively. At least three independent processing batches were manufactured per formulation under identical operational conditions. This preliminary step was essential to confirm recipe reproducibility and batch-to-batch consistency in terms of physical appearance, taste, and color, as well as moisture content and water activity, which were routinely evaluated across all produced batches.

The homogenization process was performed using a high-speed domestic homogenizer (Heinner, model HBL-1000XMC, 1000 W, Heinner, Bucharest, Romania) at high speed, for 2 min to obtain a smooth and homogenous mixture. The blend puree was spread in plastic food trays to obtain a uniform thickness (spreading thickness 4.50 ± 0.25 mm) [37] and dried in a dehydrator for fruits and vegetables (Biovita model Deluxe-6, Biovita, Cluj-Napoca, Romania).

The drying parameters were established after several attempts. The choice of the appropriate temperature–time relationship has an important role in terms of the nutritional quality of the rolls, but also of their sensory properties. A low temperature allows the preservation of most nutrients and, correlated with a long drying time, results in obtaining the desired textural and color attributes for fruit rolls.

Therefore, the fine layers of fruit puree (4.50 ± 0.25 mm) were dried at 60 °C for 6 h, in order to obtain the fruit sheets with a water activity (a_w_) value below 0.50, suitable for product stability [41]. A cooling step (20 °C) was required before cutting into rolls of a certain width.

The rolls thus obtained were packed in Kraft paper bags with windows and re-sealable zipper seals and stored properly in a dry place (20 °C), away from light and source of heat, until further analysis performed within 24−48 h.

**Figure 1:**
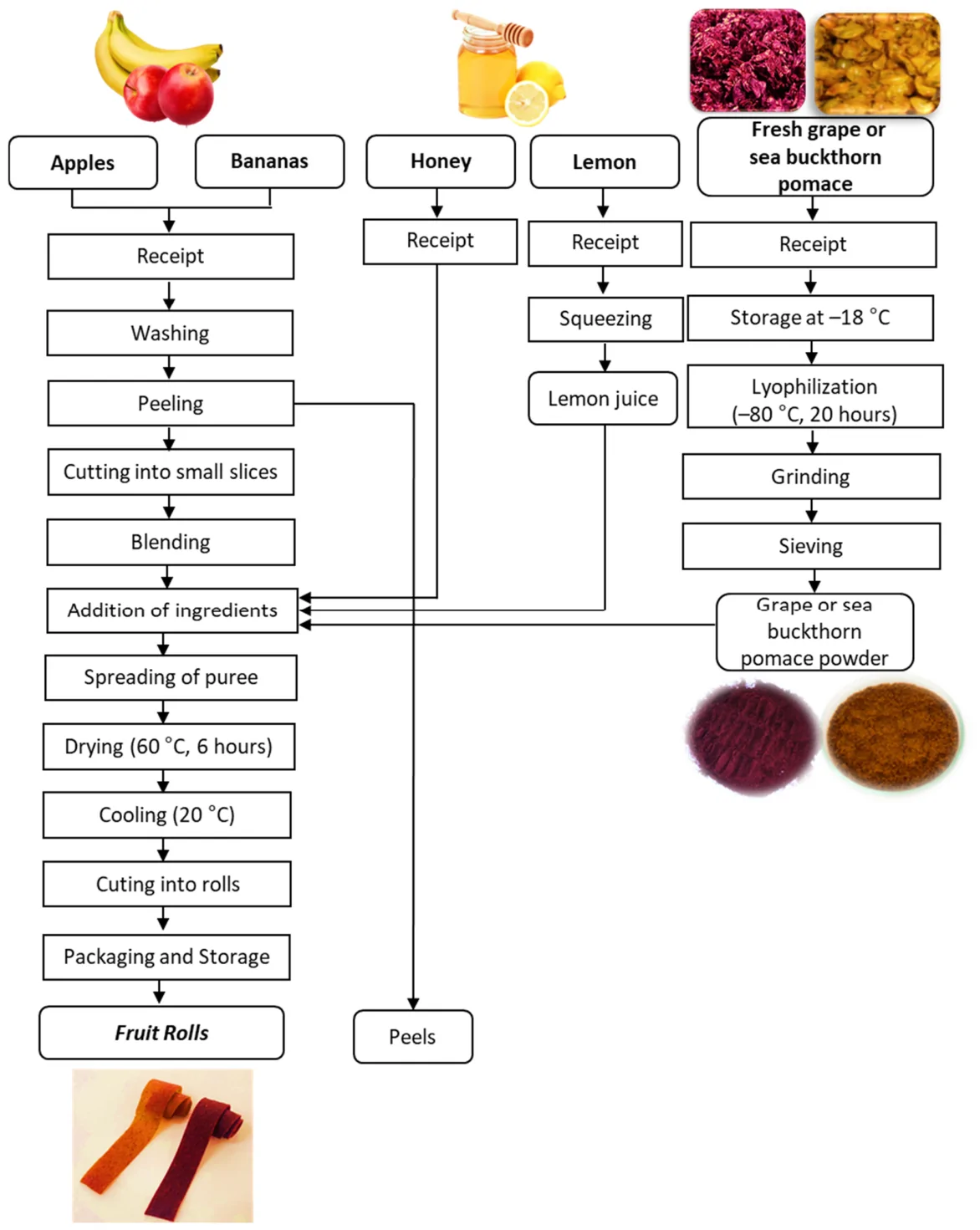
Preparation of fruit rolls enriched with freeze-dried pomace powders.

The representation of the preparation process is shown in Figure 1 (see also an illustration in Figure S1 from Supplementary Materials).

### 2.4. Characterization of Freeze-Dried Pomace Powders and Developed Fruit Rolls

Several physicochemical analyses were performed for the freeze-dried grape and sea buckthorn pomace powders and the dehydrated fruit rolls: moisture content, water activity, pH, total dissolved solids (TDS), and titratable acidity (TA). Also, the total carotenoids content (TCC), total phenolic content (TPC), and CIE L*a*b* color values were determined.

The moisture content was established using a Moisture Analyser (KERN MLB 50-3, KERN & SOHN GmbH, Bensheim, Germany) [63].

Additionally, water activity (a_w_) was measured in triplicate using a Psychrometer and Water Activity Analyzer (Rotronic HygroPalm HP23-AW-A, Rotronic Instrument Corp., New York, NY, USA).

The fruit rolls samples were ground using a householder grinder (Heinner, model HCG-150SS, 1000 W, Heinner, Bucharest, Romania) in order to obtain fine particles before extraction with the appropriate solvent for each determination, as detailed below.

For the analysis of pH, TDS and TA, the pomace powders were diluted with distillated water (1:10 *w*/*v*) and extracted for 60 min at 20 °C. The solutions were filtered through filter paper (Whatman No:4). A Thermo Scientific™ Orion™ Versa Star Pro™ Multiparameter Benchtop Meter (Thermo Fisher Scientific, Waltham, MA, USA) was used for the pH and TDS analysis. The titratable acidity (TA) was measured using the titrimetric method. The aqueous solution of pomace powder was titrated using 0.1 N NaOH solution until the pH reached the 8.2 value. The results were expressed as grams of citric acid (CA) per 100 g dry matter (d.m.) [47].

The total carotenoids were quantified according to the method described by Sharma et al. with modifications concerning the solvent mixture used for extraction (acetone: water, 4/1, *v*/*v*) using a magnetic stirrer (Nahita Blue, model 692, Auxilab S.L., Navarre, Spain). The absorbance at 470 nm (A_470_) was recorded using a UV–Vis Spectrophotometer (Shimadzu UV-1280, Kyoto, Japan) [64] and the carotenoid content was calculated using following the equation:

$$Carotenoid\;content\;[mg/g]=\frac{A_{470}\times 10^{6}}{E_{0}\times 100\times d}$$

where E_0_ represents the specific coefficient of lutein (E_0_ = 2000) and d is the cuvette thickness (d = 1 cm for spectrophotometer cell) [64].

The total phenolic content (TPC) was evaluated using the Folin–Ciocalteu method described by Li et al. [65]. In this view, hydroalcoholic extracts (ethanol: water 1:1, *v*/*v*) were prepared (sample: solvent 1:10 *w*/*v*) using ultrasound-assisted extraction (Digital Pro Ultrasonic PS-10A] [15,59]. Based on gallic acid calibration curve with R^2^ = 0.996, the results were expressed as mg gallic acid equivalents (GAE)/g of dried sample. Absorbance determination was made spectrophotometrically, at 765 nm.

Also, the colorimetric data (L*, a* and b*) were determined both for the freeze-dried pomaces and the fruit rolls using ColorWiz Cam mobile application (v1.0.0, StellarNet Inc., Tampa, FL, USA) on a smartphone camera setup [66]. The choice to use a mobile-device-based application instead of a conventional spectrophotometer was a deliberate decision rooted in a sustainable, cost-effective, and accessible alternative that yields consistent results. The device camera was mounted on a tripod at a fixed vertical distance of 15 cm directly above the samples. The samples were placed on a uniform, non-reflective white paper background sheet to ensure reproducible reflectance and contrast, under constant, diffuse neutral-white laboratory LED lighting (~5000 K) positioned at an angle to eliminate specular glare and shadows. Readings were taken in triplicate at three distinct locations across the sample surface to ensure accuracy. Data acquisition was fully automated: the software evaluated the sample surface and exported a standardized digital PDF analytical report. From this digital report, the CIELAB color space coordinates (L*, a*, b*) were extracted for statistical analysis. Additionally, the hue angle was calculated as arctan(b*/a*), defining the qualitative color attribute of the sample relative to the CIELAB color space. To comprehensively evaluate the overall color shift caused by pomace enrichment, the total color difference (ΔE*) between the formulated fruit rolls and the control sample was calculated using ChromaChecker ΔE Calculator [67] based on the standard CIELAB 1976 equation:

$${∆E}_{a,b}^{*}=\sqrt{{\left({∆L}^{*}\right)}^{2}+{\left({∆a}^{*}\right)}^{2}+{\left({∆b}^{*}\right)}^{2}}$$

where ΔL*, Δa*, and Δb* represent the differences between the formulated fruit roll samples and the control for L*, a*, and b* coordinates.

Using both metrics (Hue and ΔE*) provides valuable information about the visual evolution of the product.

To align with resource efficiency and environmental considerations, one representative batch per formulation was selected for the analytical determinations. The measurements for all determinations were performed on three distinct samples taken from a single formulation batch, with each sample measured in triplicate, the results being reported as average values.

Solvents (acetone, ethanol) and other reagents (Folin–Ciocalteu reagent, sodium carbonate, gallic acid) were of analytical grade and were purchased from Sigma Aldrich Chemical Company (Darmstadt, Germany).

### 2.5. Sensory Evaluation of Developed Fruit Rolls Using 7-Point Hedonic Scale

The sensory evaluation protocol was divided into two distinct phases: a preliminary consumer survey (*n* = 50) distributed via email, followed by an in-person sensory profile assessment performed by untrained student panelists (*n* = 20).

In the first step, a qualitative sensory study was conducted on a representative sample of 50 participants. They were informed by email that the fruit rolls were prepared from fruit puree by adding various amounts of freeze-dried pomace powder. The people who took part in the market study belong to all age groups (6% under 18 years, 48% between 18–35 years old, 26% between 35–55 years old, and 20% over 55 years old); they mainly came from the urban environment, most of them being students in a percentage of 42% (all in the 18–35 years old group). They agreed to participate in the aforementioned research. The 6-questions survey, including the response options, is available in the Supplementary Materials (Survey S1. Questionnaire and results of questionnaire data). From the request addressed to participants to the survey, namely: “Please express the first thing (in one word) that cross your mind about the product”, a qualitative sensory study using Word Association Test was applied. This technique represents an efficient and rapid method for obtaining information on consumer perceptions of local foods and conventional and functional foods, respectively, proving to be a useful tool in the domain of food research [68,69]. A word cloud was created using the Word Art web-based free program [70].

The evaluated sensory attributes were appearance, color, taste–aroma, sweetness, and texture using a 7-point hedonic scale: “7 = like very much”, “6 = like moderately”, “5 = like slightly”, “4 = neither like nor dislike”, “3 = dislike slightly”, “2 = dislike moderately”, and “1 = dislike very much”. The same scale was used for the degree of global satisfaction of our product [69,71].

The untrained panelists, aged between 20 and 52 years, consisted of twenty members from the Bachelor and Master students. Informed consent was obtained from all panelists prior to evaluation, confirming voluntary participation and absence of food allergies, in accordance with institutional ethical guidelines. The sensory analysis panelists and the questionnaire respondents represented two entirely different participant groups. Participants received a plate containing the samples, a glass of water, and an evaluation sheet. To ensure blind testing conditions, all samples were coded with randomized 3-digit numbers and presented in identical neutral containers. The sample presentation order was fully randomized and counterbalanced across participants to eliminate order and carryover bias. Adequate space was given to handle the samples and the questionnaire, and the evaluation time was not constrained.

Sensory profile diagrams were plotted based on the statistically analyzed data using Microsoft EXCEL 2010.

### 2.6. Statistical Analysis

Physicochemical and chemical analyses were performed in triplicates, and the results are expressed as mean of each sample ± standard deviation (SD). Consequently, all statistical analyses reflect analytical sub-sampling replication within a representative production batch rather than variation across independent processing runs.

Data were analyzed using one-way analysis of variance (ANOVA) followed by Tukey’s post hoc test for means comparison at a significance level (*p* < 0.05). Origin 2024 10.1.0.170 Academic software (OriginLab Corporation, Northampton, MA, USA) was used for the statistical analysis.

## 3. Results and Discussion

### 3.1. Characterization of Freeze-Dried Pomace Powders

The measured physicochemical and chemical parameters for the freeze-dried pomace powders used in the present study are shown in Table 1.

The statistical analysis indicates significant differences between pomace powders characteristics (*p* < 0.05). FD-SBPP and FD-GPP showed similar water activities, but the moisture content is lower in the case of FD-SBPP. A smaller pH value and a higher titratable acidity, were found in sea buckthorn dried marc, respectively, compared to grape pomace. It is known that the sea buckthorn fruits and implicitly the derived pomace are characterized by relatively high acidity. The pH of FD-GPP (3.63) was similar to that reported by Yeler et al. (3.54) for the pomace from an indigenous Turkish grape variety and the titratable acidity was higher (3.97) [72].

A significantly higher content of total phenolic compounds (*p* < 0.05) was found in FD-SBPP compared to FD-GPP. The obtained TPC values are slightly higher than those reported by some studies: 33.51 mg GAE/g for the freeze-dried sea buckthorn berries [64], 28.56 ± 0.24 mg GAE/g d.m. for the sea buckthorn pomace [22], 17.07 mg GAE/g for microwaved-dried grape pomace (*Fetească neagră* variety) [14] or 19.38 ± 0.12 mg GAE/g for the lyophilized grape pomace wine [73].

The two freeze-dried pomaces each exhibited distinct color profiles based on their CIEL*a*b* values. FD-SBPP featured a light color profile, characterized by a high lightness value L* (68.04 ± 0.70) together with prominent yellowness b* (53.59 ± 0.78). The values are comparable with the data reported in the literature, but slightly different. Zhou et al. [22] reported maximum L* (57.59 ± 0.05) and b* (61.61 ± 0.10) for the sea buckthorn pomace obtained by vacuum freeze drying, but for a moisture content below 1%, much lower than the FD-SBPP from the present study. FD-GPP displayed a distinctly darker profile, with a low lightness value L* (29.47 ± 0.87) and strong redness a* (30.66 ± 1.73). Stoica et al. [44] reported lower values for L* (23.36 ± 0.16) and a* (10.37 ± 0.05), for the conventional-dried grape pomace powder compared with the values obtained in our study.

### 3.2. Characterization of Prepared Fruit Rolln

The developed fruit rolls were analyzed in terms of some physicochemical, chemical, and colorimetric characteristics (Table 2).

Fruit rolls dried under the same conditions showed similar moisture content (between 15.19 and 16.10) and water activity (0.48–0.49) comparable with those described in the literature [37,41,54]. The maintained low water activity (<0.50) is particularly crucial, as it limits free water availability, suppressing enzymatic degradation and preserving the structural integrity of the carbohydrate matrix. Although explicit shelf-life trials were not conducted in the present work, there is strong literature evidence that low water activity is a key parameter ensuring microbiological stability during storage [37,41]. For instance, Vázquez-Sánchez et al. [41] reported that fruit leathers stored for 12 weeks at 25 °C exhibited no visible mold growth or significant parameter changes, directly attributing this stability to low a_w_ and pH values.

The pH of the fruit rolls slightly decreased from 5.97 to 5.63, when FD-GPP was added. As well, a significant decrease in pH from 5.97 to 5.05 was observed when dehydrated fruit rolls were obtained with the addition of FD-SBPP, due to the higher acidity of sea buckthorn pomace. This increased acidity impacts taste perception and overall flavor balance.

The addition of freeze-dried pomace powders improved the bioactive profile of the samples by significantly increasing their content of phenolic compounds and carotenoids. Specifically, carotenoid content increased from 5.92 mg/g d.m. in the control to 9.12 and 17.05 mg/g d.m. with the addition of FD-GPP and FD-SBPP, respectively. TPC value increased from 59.62 to 86.54 mg GAE/g d.m. in the case of fruit rolls enriched with FD-GPP. The highest intensification in TPC was observed for fruit rolls with FD-SBPP addition (95.77 mg GAE/g d.m.), in correlation with the TPC content of the powder. This strong correlation between raw pomace composition and final product bioactive content demonstrates that freeze-drying effectively protects phenolics and carotenoids prior to incorporation into the fruit roll matrix.

As expected, the color of prepared samples differed significantly from the control. CIE L*a*b* values for the control apple and banana leather were 61.05 ± 2.59 for lightness, 10.99 ± 0.88 for redness and 32.96 ± 1.45 for yellowness. The addition of FD-SBPP enhanced the yellowness level (b* from 32.96 to 41.21), while keeping the Hue identical to the control. CIEL*a*b* values of fruit rolls completely change when FD-GPP was added, shifting strongly toward the redness, implicitly important decreases in lightness, yellowness, and hue, respectively.

The total color difference of ΔE* values clearly demonstrated the varying impact of the two pomace types on the visual profile of the fruit rolls. It is known that values between 2 and 10 indicate a perceptible color difference that is easily noticed by an untrained observer, while values exceeding 10 represent a profound color transformation. The fruit rolls enriched with FD-SBPP exhibited a value of 9.64. This is a clearly noticeable color difference compared to the control, but it did not completely transform into a different color spectrum. Thus, the addition of FD-SBPP intensified and brightened the existing yellow-brown matrix to a more vibrant color. The incorporation of FD-GPP led to a complete color transformation, resulting in a value of 36.57, giving to the final fruit rolls a completely new color from the purple spectrum. Both formulations produced a significant visual differentiation compared to the control. These color changes reflect that the polyphenols and the carotenoids from pomaces are very well incorporated into the pectin network of the fruit.

The observed variations in moisture content, pH, total phenols, and carotenoids directly reflect the distinct chemical composition of the added pomace powders and their interaction with the drying process. While these physical and compositional traits confirm the success of pomace incorporation, other important quality attributes (e.g., instrumental texture profile such as chewiness, stickiness, and elasticity), are equally vital in governing consumer acceptance and require continued investigation.

### 3.3. Sensory Evaluation of Dehydrated Fruit Rolls

Following the qualitative sensory study using Word Association Test, the fifty participants who responded to our survey regarding the first impression, in one word, designated most frequently words like “tasty”, “interesting”, or “delicious” (designated by 30% of participants). Other associated words were “healthy”, “dietary”, “creative”, “innovative”, and “new” (attributed by 32% of participants). All assigned words and the percentage of participants who designated them are detailed in Table S2 (Supplementary Materials). Similar words such as “healthy”, “interesting”, or “novel” were reported by Vázquez-Sánchez et al. for their apple leathers enriched with acachul powder [41].

**Figure 2:**
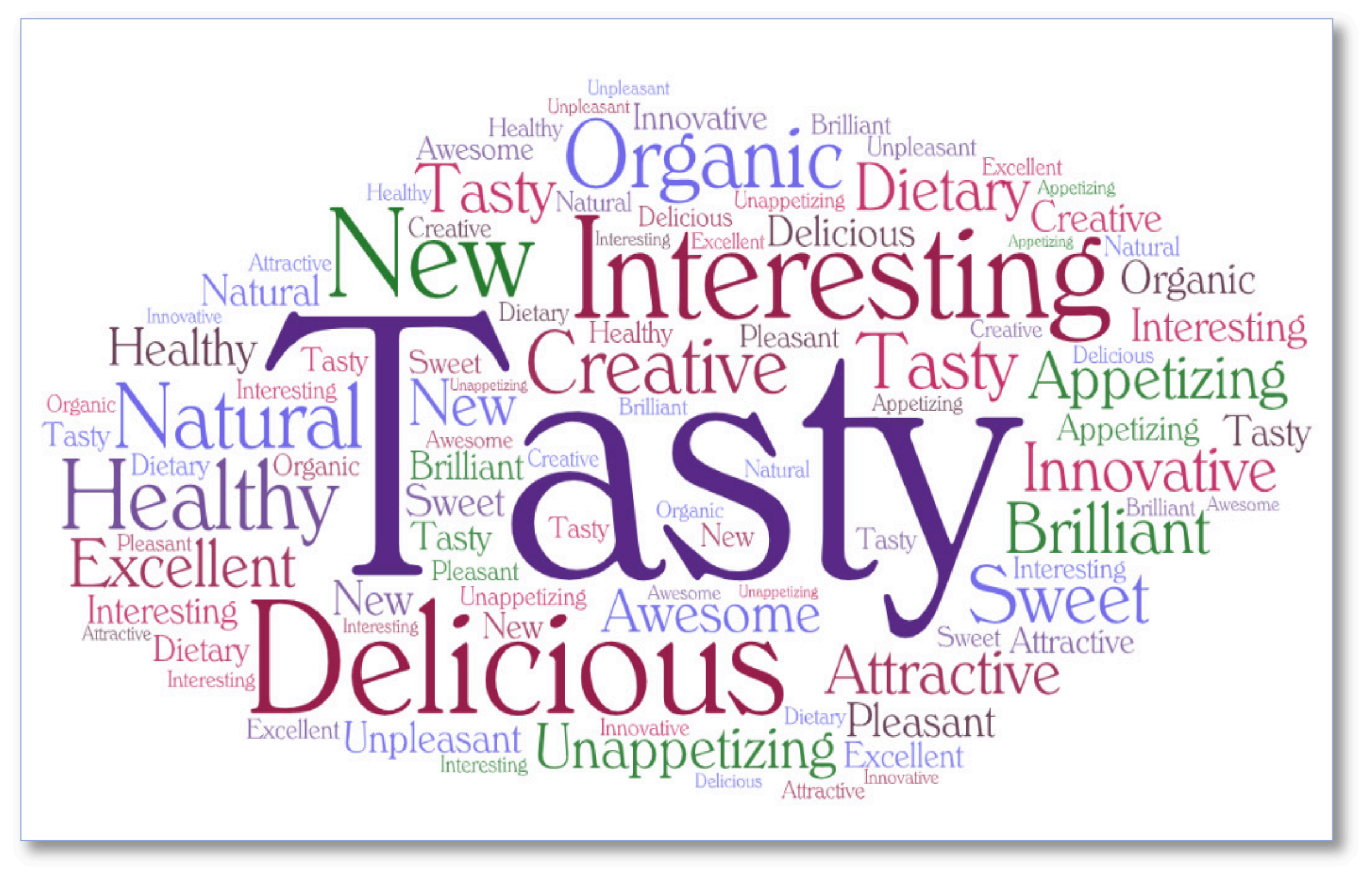
Word cloud constituted of the different words mentioned by the participants of the survey (*n* = 50).

The word cloud constituted of the different words mentioned by the participants of the survey (*n* = 50) through the qualitative sensory study presented in Figure 2.

**Figure 3:**
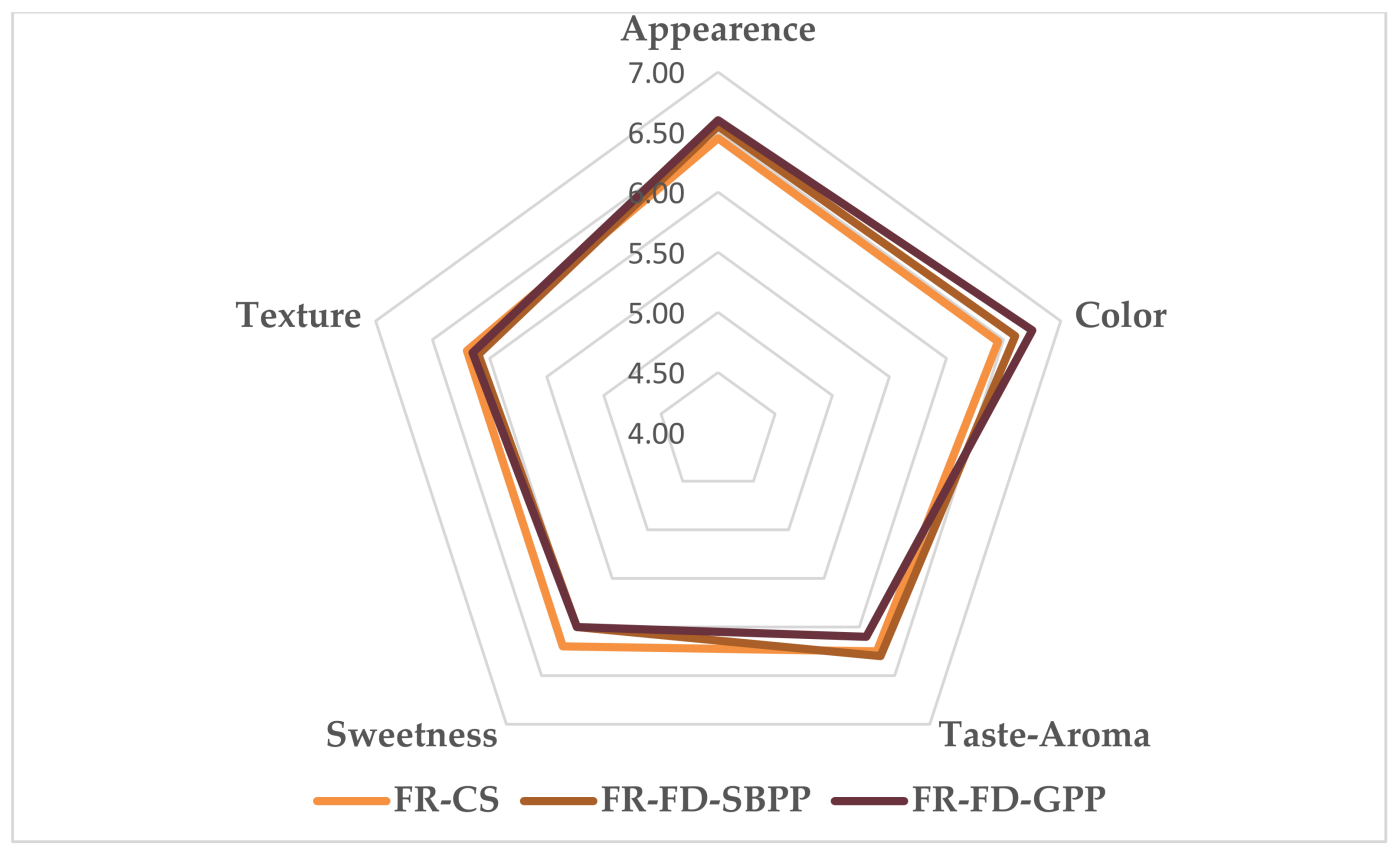
Sensory profile of fruit rolls by attribute (FR-CS—fruit rolls control sample; FR-FD-SBPP—fruit rolls with freeze-dried sea buckthorn pomace powder; FR-FD-GPP—fruit rolls with freeze-dried grape pomace powder).

The results of the descriptive analysis of the sensory evaluation of the dehydrated fruit rolls with lyophilized pomace revealed slightly different average scores compared to the control sample (Figure 3). For appearance and color, fruit rolls with FD-GPP received the highest scores, followed by samples with FD-SBPP, suggesting that pomace powders addition improved the visual appeal of the product. Color remains a critical challenge in by-product valorization, as documented by previous literature where the incorporation of just 1.5% wild bilberry and blackcurrant pomace powders resulted in an excessively dark coloration that significantly reduced sensory acceptance [47]. In contrast, our findings demonstrated that even at a higher incorporation level of 3%, the fruit rolls maintained an appealing visual profile, receiving sensory color scores comparable to the control sample.

Fruit rolls enriched with FD-SBPP showed favorable taste–aroma characteristics, on the other hand influencing sweetness due to the sour taste specific to sea buckthorn. This acidic note provided a distinctive, well-accepted fruitiness without overly masking the sweet base. This trend aligns closely with the findings reported in bakery matrices; for instance, when wheat bread was fortified with sea buckthorn pomace powder, the resulting samples exhibited a distinct sea buckthorn aroma and taste, and for higher incorporation percentages were characterized by pronounced acidity and bitterness notes [25]. The results confirm that across different food matrices—whether structured biopolymer leathers or baked goods—the inherent organic acids and polyphenols of sea buckthorn consistently modulate sweetness perception and dictate overall sensory compliance. Regarding grape pomace powder formulation, the taste–aroma scores were slightly lower than those of the control and sea buckthorn-enriched samples. This can be attributed to the slight astringency and phenolic bitterness inherent to grape pomace, which subtly affected overall flavor.

The control sample (FR-CS) obtained the highest score for sweetness and texture, and by adding pomace powder it can be noted that these attributes are slightly reduced. From the texture perspective, the shift in product quality is driven by the structural properties of the pomace powders. Incorporating insoluble fiber-rich pomace disrupts the continuous pectin–carbohydrate gel matrix formed by the apple–banana base, conducive to a less cohesive, or grittier, texture. It is known that depending on the proportion of powder added, a decrease in the texture of the fruit leathers may result [41,47,74].

In term of global satisfaction, all samples obtained a score above 6.00, increasing slightly from 6.05 for the control sample to 6.15 and 6.20 for the samples formulated with FD-SBPP and FD-GPP, respectively, corresponding approximately to a “like moderately” score. The addition of 3% sea buckthorn or grape pomace powder did not negatively affect overall satisfaction, suggesting that these ingredients are suitable for maintaining good product acceptability.

While the initial physicochemical and sensory evaluations demonstrate the potential of these fruit rolls as alternative snacks, certain technological limitations of the present study must be acknowledged. Specifically, instrumental texture profile analysis, long-term quality maintenance, and microbiological stability during extended storage remain to be systematically evaluated.

## 4. Conclusions

This study successfully demonstrates a circular economy approach to food sustainability by upcycling industrial beverage by-products into potentially functional, value-added food ingredients. By integrating freeze-dried sea buckthorn and grape pomaces, the developed new fruit rolls were characterized by significant enrichments in total polyphenols and carotenoids compared to the control. Beyond fortification, colorimetric analysis confirmed that the lyophilized pomaces served as effective natural colorants, imparting attractive orange and dark purple hues that achieved high panelist acceptance during sensory evaluation. Formulated entirely without artificial preservatives, synthetic colorants, or other chemical additives, these clean-label fruit rolls represent a tasty alternative to conventional sweet snacks. By valorizing both defective fruit raw materials and beverage industry by-products, this work addresses critical food waste management challenges and supports a circular food supply chain.

To build upon these preliminary findings, future research efforts will focus on detailed chromatographic profiling of phenolic compounds (specifically targeting flavonoid and anthocyanin profiles), alongside comprehensive nutritional evaluation and wider consumer sensory evaluation. Furthermore, investigating storage stability and shelf-life will be critical to validating sensory persistence and establishing commercial viability. Beyond the current formulations, this framework can be expanded by exploring the functional incorporation of other berry by-products, such as redcurrant, blackcurrant, cranberry, and blackberry pomaces.

Finally, to achieve a fully zero-waste production cycle, waste generated during processing will be converted into stable, dehydrated functional powders. Co-utilizing these fruit residues with complementary organic waste, such as eggshells and spent coffee grounds, represents a promising strategy for developing eco-friendly soil biostimulants or biofertilizers to support plant growth and development (Supplementary Materials, Figure S2). This cascading valorization approach directly reinforces our commitment to total waste recovery and circular bioeconomy principles.

## Figures and Tables

**Table 1:** Physicochemical and chemical characteristics of freeze-dried pomace powders.

Characteristic		FD-SBPP	FD-GPP
Moisture content [%]		7.87 ± 0.31 ^b^	8.88 ± 0.23 ^a^
Water activity		0.32 ± 0.02 ^a^	0.35 ± 0.01 ^a^
pH of aqueous extract		3.21 ± 0.05 ^b^	3.63 ± 0.08 ^a^
TDS of aqueous extract [ppm]		737.50 ± 0.43 ^b^	1181.23 ± 1.80 ^a^
Titratable acidity [g CA/100 g d.m.]		5.13 ± 0.24 ^a^	3.97 ± 0.19 ^b^
Total phenolic content [mg GAE/g d.m.]		35.37 ± 1.90 ^a^	21.01 ± 0.23 ^b^
Carotenoid content [mg/g d.m.]		17.20 ± 0.21 ^a^	10.52 ± 0.12 ^b^
CIEL*a*b*	L*	68.04 ± 0.70	29.47 ± 0.87
	a*	3.95 ± 0.14	30.66 ± 1.73
	b*	53.59 ± 0.78	11.41 ± 0.93
Hue		85.78 ± 0.84	20.30 ± 0.89

The values are expressed as mean ± standard deviation (SD). Values with different superscript letters in the same row are significantly different at *p* < 0.05 according to Tukey’s test.

**Table 2:** Comparative parameters of fruit rolls prepared with freeze-dried pomace powders addition and control formulation.

Characteristic		Fruit Rolls Without Pomace (Control)	Fruit Rolls with FD-SBPP Addition	Fruit Rolls with FD-GPP Addition
Moisture content [%]		16.10 ± 0.21 ^a^	15.65 ± 0.23 ^ab^	15.19 ± 0.18 ^b^
Water activity		0.48 ± 0.002 ^a^	0.48 ± 0.002 ^a^	0.49 ± 0.004 ^a^
pH of aqueous extract		5.97 ± 0.07 ^a^	5.05 ± 0.05 ^c^	5.63 ± 0.09 ^b^
Titratable acidity [g CA/100 g d.m.]		2.35 ± 0.03 ^c^	3.23 ± 0.07 ^a^	2.77 ± 0.05 ^b^
Total phenolic content [mg GAE/g d.m.]		59.62 ± 1.58 ^c^	95.77 ± 3.93 ^a^	86.54 ± 3.68 ^b^
Carotenoid content [mg/g d.m.]		5.92 ± 0.38 ^c^	17.05 ± 0.66 ^a^	9.12 ± 0.26 ^b^
CIEL*a*b*	L*	61.05 ± 2.59 ^a^	56.70 ± 1.76 ^b^	34.07 ± 1.78 ^b^
	a*	10.99 ± 0.88 ^b^	13.45 ± 0.97 ^b^	24.62 ± 0.70 ^a^
	b*	32.96 ± 1.45 ^b^	41.21 ± 3.45 ^a^	12.37 ± 0.46 ^c^
Hue		71.56 ± 1.07 ^a^	71.84 ± 1.69 ^a^	26.56 ± 0.50 ^b^
ΔE*		-	9.64 ± 1.80 ^b^	36.57 ± 0.98 ^a^

The values are expressed as mean ± standard deviation (SD). Values with different superscript letters in the same row are significantly different at *p* < 0.05 according to Tukey’s test.

## Data Availability

The original contributions presented in this study are included in the article/Supplementary Materials. Further inquiries can be directed to the corresponding author.

## Supplementary Materials

The following supporting information can be downloaded at: https://www.mdpi.com/article/10.3390/foods15173059/s1, Figure S1. Illustration of the preparation process of dehydrates fruit rolls; Table S1. Initial trials in order to find the optimal formulation; Survey S1. Questionnaire and results of questionnaire data; Table S2. All words attributed to the participants of the survey; Figure S2. Potential sustainable approach for an appropriate management of wastes generated by the obtention of fruit rolls.
