# Supplementary material for: Development, Physicochemical, and Sensory Evaluation of Naturally Formulated Dehydrated Fruits Rolls Enriched with Sea Buckthorn and Grape Freeze-Dried Pomace Powders
Source: Foods. 2026 Aug 28;15(17):3059. doi: 10.3390/foods15173059 (PMC13564743; doi:10.3390/foods15173059)
Supplement: Supplementary file 1 [file foods-15-03059-s001.zip › foods-4489924-supplementary.pdf]

Development, Physicochemical, and Sensory Evaluation of Naturally Formulated Dehydrated Fruits Rolls Enriched with Sea Buckthorn and Grape Freeze-Dried Pomace Powders

Luminița Grosu, Oana-Irina Patriciu, Irina-Claudia Alexa * and Adriana-Luminița Fînaru

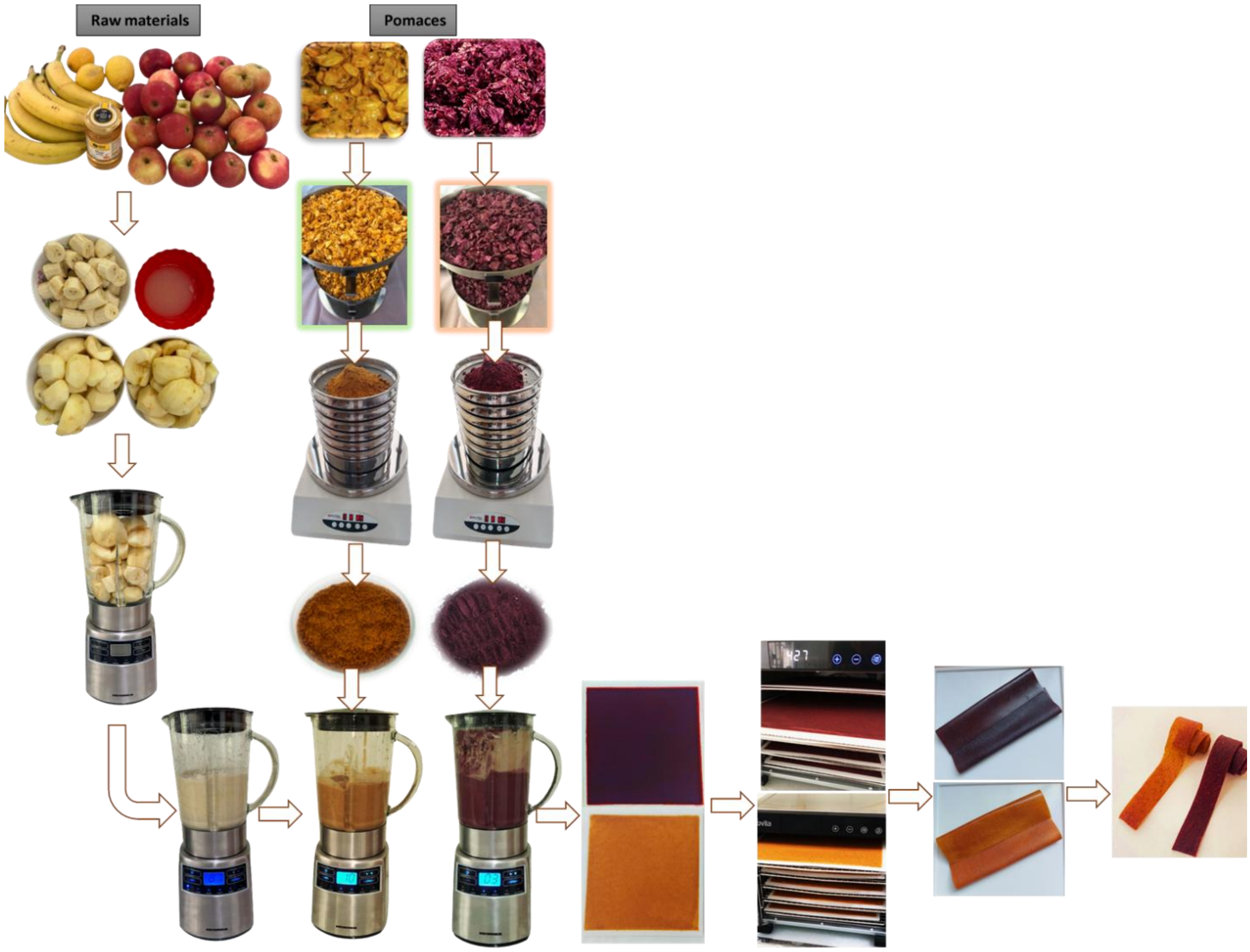

Figure S1. Illustration of the preparation process of dehydrates fruit rolls.

Table S1. Initial trials in order to find the optimal formulation

Trial	Formulations / ratios tested	Informal sensory and physical observations
**Base puree ratio** *(Peeled apples : bananas)*	1:1 (*w/w*)	- High banana flavor dominated the profile<br>- Product was slightly too soft and sticky during dehydration
	3:1 (*w/w*)	- Excessively tart base profile (Idared variety is a sour apple)<br>- A delayed dehydration, leading to an unbalanced sheet structure
	2:1 (*w/w*)	- Optimal balance of sweetness and acidity<br>- Provided good texture, structural integrity, and rolling properties
**Additive levels** *(honey and lemon juice)*	5% honey (w/w) + 2% lemon juice (w/w)	- Product was too sweet
	2% honey (w/w) + 2% lemon juice (w/w)	- Harmonized sweet-sour balance<br>- Enhanced natural fruit color preservation<br>- Maintained proper drying
**Pomace powder incorporation** *(sea buckthorn / grape)*	6% (w/w)	- Astringency mouthfeel (in the case of FD-GPP) or sourness (in the case of FD-SBPP)<br>- Dark color in the case of FD-GPP
	3% (w/w)	- Suitable color enhancement<br>- Distinct functional flavor profile without grittiness

Survey S1. QUESTIONNAIRE AND RESULTS OF QUESTIONNAIRE DATA

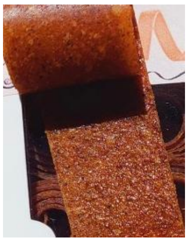

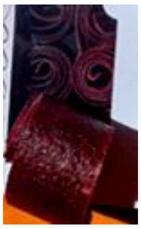

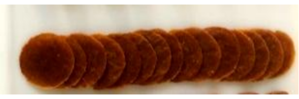

Dear Madame/Sir,

Please read the following statements and tick the boxes below if you agree.

☐ I agree to take part in the above research.

☐ I understand that my agreement to participate in this research does not affect my legal rights.

We are conducting an opinion poll on new products.

The presented products are obtained from fruits puree (apples and bananas), by drying using a dehydrator (at low temperatures, for 6-7 hours). Various powders of freeze-dried by-products (pomace) resulting from the production of sea buckthorn juice and wine were added. Pomace (also called marc) is the pulpy residue of skins, seeds, and stems left over after pressing fruits for their juice or oil and contain various bioactive compounds with health benefits.

Please be kind and answer to this questionnaire regarding the food product briefly described and presented in images.

BEFORE STARTING THE QUESTIONNAIRE, CONSIDERING THE ABOVE DESCRIPTION PLEASE EXPRESS THE FIRST THING (IN ONE WORD) THAT CROSS YOUR MIND ABOUT THE PRODUCT.

1. Have you ever heard of dehydrated fruit leather or rolls?
□ Yes □ No

2. Have you ever eaten dehydrated fruit rolls?
□ Yes □ No

3. Do you think this product is also suitable for children?
□ Yes □ No

4. What is your opinion about the fact that this product valorizes a series of by-products?
□ Agree □ Neither Agree Nor Disagree □ Disagree

5. Mark three important characteristics that, in your opinion, fruit rolls should have.
□ Aspect □ Consistency □ Color □ Flavor □ Taste □ Shelf life

6. Please suggest which way of presentation (rolls or chips) seems more suitable/attractive for the product, in your opinion?
□ Rolls □ Chips

Identification data

Gender: ☐ Male ☐ Female ☐ Other ☐ Prefer not to say

Age: ☐ under 18 years ☐ 18-35 years old ☐ 35-55 years old ☐ over 55 years old

General area where you live: ☐ Urban ☐ Rural

Thank you for your time!

78% of the 50 respondents to this questionnaire had heard of fruit leather or rolls and only 52% reported having consumed them. The product seemed suitable for children by 44 of those surveyed (88%), and they even indicated it as a substitute for sweet snacks or candy.

Graphics containing the responses for the questions 4, 5 and 6 respectively, are presented below.

The product in roll form was preferred by 72%, therefore, our study targeted the dehydrated products presented in roll form.

The characteristics that were most checked by respondents were taste (34/50), color (28/50) and equally aspect and shelf life (24/50).

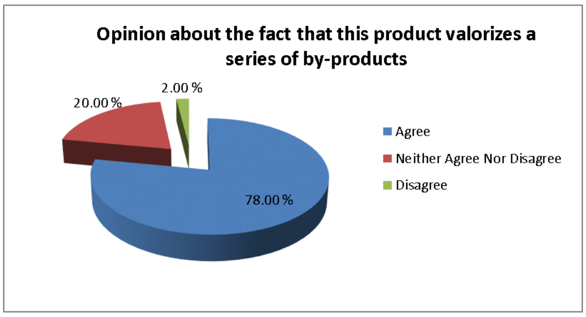

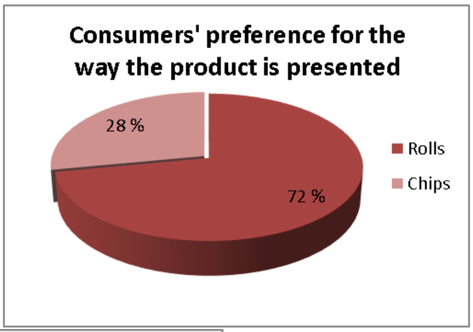

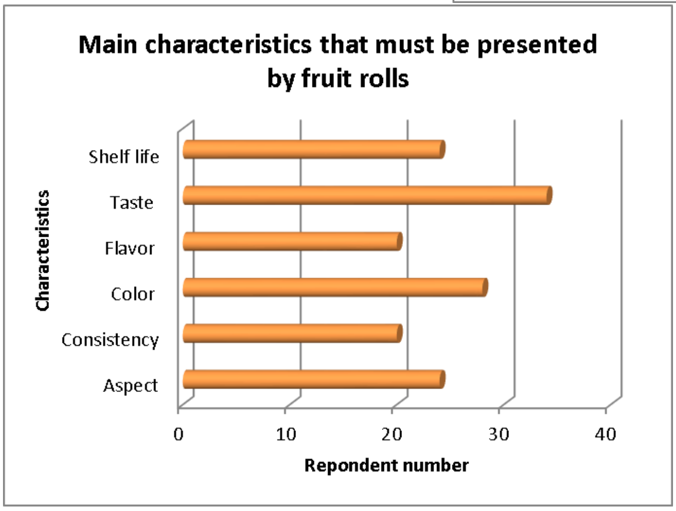

Table S2. All words attributed by the participants of the survey

Entry	Words	Number of participants	Percentage
1	Tasty	8	16%
2	Delicious	6	12%
3	Interesting	6	12%
4	Healthy	4	8%
5	Dietary	4	8%
6	Creative	3	6%
7	Innovative	3	6%
8	Natural	3	6%
9	Organic	2	4%
10	New	2	4%
11	Sweet	1	2%
12	Awesome	1	2%
13	Brilliant	1	2%
14	Excellent	1	2%
15	Pleasant	1	2%
16	Attractive	1	2%
17	Appetizing	1	2%
18	Unpleasant	1	2%
19	Unappetizing	1	2%
	Total	**50**	**100%**

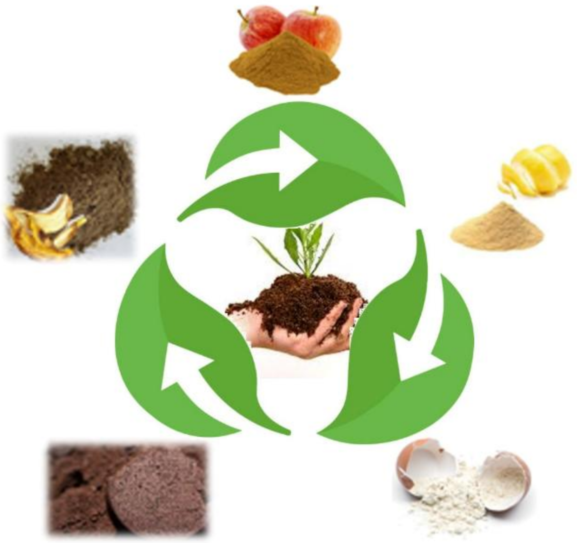

Figure S2. Potential sustainable approach for an appropriate management of wastes generated by the obtention of fruit rolls.
